# Cryptic Diversity and Ecological Overlap in *Sporothrix schenckii*: Insights from Multilocus Phylogenetics of Clinical and Environmental Isolates

**DOI:** 10.3390/jof11110759

**Published:** 2025-10-22

**Authors:** Carolina Brunner-Mendoza, Anderson Messias Rodrigues, Esperanza Duarte-Escalante, María del Rocío Reyes-Montes, Amelia Pérez-Mejía, Hortensia Navarro-Barranco, María del Carmen Calderón-Ezquerro, Conchita Toriello

**Affiliations:** 1 Departamento de Microbiología y Parasitología, Facultad de Medicina, Universidad Nacional Autónoma de México, Ciudad de Mexico 04510, Mexico; brunner.carolina@facmed.unam.mx (C.B.-M.);; 2 Laboratory of Emerging Fungal Pathogens, Department of Microbiology, Immunology, and Parasitology, Discipline of Cellular Biology, Federal University of São Paulo (UNIFESP), São Paulo 04023-062, Brazil; amrodrigues.amr@gmail.com; 3 National Institute of Science and Technology in Human Pathogenic Fungi, São Paulo 04023-062, Brazil; 4 Departamento de Ciencias de Ambientales, Instituto de Ciencias de la Atmósfera y Cambio Climático, Universidad Nacional Autónoma de México, Ciudad de Mexico 04510, Mexico; mclce@atmosfera.unam.mx

**Keywords:** *Sporothrix schenckii*, MLSA (multilocus sequence analysis), genetic diversity

## Abstract

*Sporothrix schenckii* is a pathogenic fungus with both clinical and environmental origins that was traditionally described as a single species but is increasingly recognized as being genetically diverse. In this study, we analyzed multiple isolates recovered from human sporotrichosis cases and environmental sources across Latin America (Mexico, Guatemala, Colombia). We conducted a polyphasic analysis of 16 isolates, integrating morphological data with multilocus sequence analysis (MLSA) targeting the internal transcribed spacer (ITS), calmodulin (*CAL*), β-tubulin (*BT2*), and translation elongation factor 1-α (*TEF*) gene regions. Phylogenetic relationships were resolved via maximum likelihood, and genetic structure was corroborated via four independent clustering methods: minimum spanning tree, principal component analysis, multidimensional scaling, and self-organizing maps. MLSA reidentified six isolates as *S. globosa* and confirmed the absence of *S. brasiliensis* in the cohort. The remaining *S. schenckii s. str.* isolates were resolved into three clades (A, B, and C). Notably, clade B (EH748, EH194, and EH257) formed a genetically divergent cluster with the highest nucleotide diversity (π = 0.03556) and was consistently segregated by all clustering algorithms. Clinical and environmental isolates were phylogenetically intermingled, supporting an active environmental reservoir for human infections. Phenotypic data, including colony size and conidial and yeast dimensions, varied but did not clearly distinguish between clinical and environmental origins. Our study provides compelling molecular evidence for a previously unrecognized, highly divergent clade within *S. schenckii s. str.*, indicative of ongoing cryptic speciation. These findings refine the taxonomy of medically important *Sporothrix* species and reveal a distinct epidemiological profile for sporotrichosis in the studied regions, separate from the *S. brasiliensis*-driven epizootic. This highlights the critical role of molecular surveillance for accurate diagnosis, treatment, and public health strategies.

## 1. Introduction

The taxonomy of the genus *Sporothrix* has undergone substantial revision with the advent of molecular phylogenetics, particularly through multilocus sequence analyses targeting loci such as calmodulin (*CAL*) and β-tubulin (*BT2*) [1]. Formerly considered a monotypic genus centered on *Sporothrix schenckii*, it is now recognized as a diverse group of phylogenetically distinct species [2]. These taxa differ markedly in geographic distribution, pathogenicity, clinical manifestations, and antifungal susceptibility, supporting their recognition as separate species rather than as members of a cryptic complex. The genus is currently structured into two major clades: a pathogenic clade, comprising the principal agents of mammalian sporotrichosis, including *S. brasiliensis*, *S. schenckii*, *S. globosa*, and *S. luriei*, and an environmental clade, which encompasses species commonly associated with soil, vegetation, and insects [3]. While typically saprobic, some environmental species, particularly those within the *S. pallida* and *S. stenoceras* complexes, have been implicated as opportunistic pathogens [4,5]. To date, *Sporothrix* includes 70 described species, reflecting remarkable ecological and biological diversity [6,7].

Sporotrichosis is a subcutaneous implantation mycosis of global relevance caused by pathogenic *Sporothrix* that affects both humans and a wide range of animals, particularly felines. Clinically, the disease most commonly presents as nodular and ulcerative lesions on the skin and subcutaneous tissues, often progressing along lymphatic pathways in its lymphocutaneous form [3]. Transmission occurs primarily through two routes. The classical sapronotic pathway involves traumatic inoculation of fungal elements from environmental sources such as contaminated soil or plant debris, posing an occupational risk to individuals engaged in agricultural or gardening activities. Alternatively, zoonotic transmission, which has become increasingly prominent in recent decades, occurs through scratches or bites from infected animals, especially domestic cats harboring *S. brasiliensis* [7]. This animal-associated route has driven major outbreaks, particularly in South America, underscoring the public health significance of feline sporotrichosis. Although less common, pulmonary sporotrichosis may also develop following the inhalation of fungal propagules [8].

In Mexico, *Sporothrix* poses a health concern primarily in rural areas, with a higher incidence observed in patients aged 0–15 years; however, research on *Sporothrix* populations in this context remains limited [9]. Studies on *S. schenckii sensu lato* isolates from Mexico have revealed distinct characteristics, including conidial length, thermotolerance, median lethal dose, and genotypic traits analyzed through random amplified polymorphic DNA (RAPD) [10]. Additionally, research has investigated the genetic diversity and subclades within *S. schenckii*, employing *CAL* and ITS of the rRNA gene for phylogenetic analysis. The observed variations in Mexican *Sporothrix* isolates may be linked to differing degrees of virulence, with this diversity potentially arising from the complex interplay of host–pathogen interactions, ecological processes, and environmental factors [11].

In this study, we investigated the phylogenetic relationships of clinical and environmental *S. schenckii s. str.* isolates collected from Mexico, Guatemala, and Colombia. We aimed to evaluate the extent of genetic diversity within the species, cluster by ecological origin, and explore phenotypic features such as colony and yeast dimensions that may correlate with genotype or host. Our results provide insight into the diversity of *S. schenckii* and contribute to the ongoing discussion regarding its taxonomy, evolution, and epidemiology. This knowledge contributes to the development of accurate treatment strategies and enhances our understanding of the epidemiology surrounding sporotrichosis.

## 2. Materials and Methods

### 2.1. Fungal Strains

The fungal strains analyzed in this study were obtained from various institutions (Table 1). At the time of isolation, strains were identified as *Sporothrix schenckii* based on conventional morphological and cultural characteristics. Because these isolates originated from different medical laboratories, detailed metadata regarding their collection (e.g., patient clinical information, type of specimen, or specific environmental sampling conditions) is unavailable. The isolates included samples from Mexican soils (*n* = 7) and clinical specimens from patients diagnosed with sporotrichosis in Mexico (*n* = 4), Guatemala (*n* = 1), and Colombia (*n* = 4) (Table 1). The strains have been preserved under different conditions, including sterile water, mineral oil, and liquid nitrogen, and are maintained in the fungal collection of the Laboratorio de Micología Básica, Departamento de Microbiología y Parasitología, Facultad de Medicina, Universidad Nacional Autónoma de México (UNAM). This collection is registered in the World Federation for Culture Collections (WFCC) under the accession number BMFM-UNAM 834.

### 2.2. Morphological and Physiological Studies

The micromorphological characteristics were determined with lactophenol cotton blue. Diagnostic characteristics were observed with an Olympus microscope (CH2). Ten conidia from 15-day-old PDA cultures at 28 °C, as described by [12] Dixon et al., 1991, of each isolate were measured (width and length) with a calibrated ocular micrometer (Olympus CH2). The conidial dimensions were subjected to analysis of variance (ANOVA, α = 0.01 and 0.05) to detect significant differences. Single conidial cultures were grown on PDA, Sabouraud dextrose agar (SDA), or corn meal agar (CMA) and incubated at 25 °C to promote sporulation. Colonies were imaged after 21 days of growth via an FNE-NX9 camera.

The assimilation of carbon sources (sucrose, raffinose) was tested in yeast nitrogen base (YNB, Sigma-Aldrich, St. Louis, MO, USA) liquid media. The test was performed in 96-well ELISA plates (Sigma-Aldrich, St. Louis, MO, USA) with 50 µL of inoculum of 2 × 10^5^ to 2 × 10^6^ and 50 µL of YNB medium at 25 °C for 10 days according to [13]. For the positive control, 100 µL of a *Cryptococcus* sp. inoculum at the same volume and concentrations was used, and 100 µL of sterile distilled water was used as the negative control.

The thermodimorphic transition was performed in 96-well microplates, with each well containing 100 μL of brain–heart infusion (BHI) medium (pH 7.2) and an inoculum of 10^6^ conidia/mL from seven-day-old yeast extract peptone dextrose (YPD) slants of each fungal isolate. The cultures were incubated at 37 °C for seven days, and the yeast forms were observed with a microscope (Olympus CH2, Tokyo, Japan).

### 2.3. DNA Extraction

The isolates were maintained on PDA and stored at 4 °C. Genomic DNA was extracted from fungal cultures by inoculating conidia from these isolates into 50 mL of YPD broth (0.2% (*w*/*v*) yeast extract, 1% peptone, 2% dextrose) in 125 mL flasks. These mixtures were shaken at 200 rpm at 27 °C for 3 to 4 days. The mycelial mat was collected by vacuum filtration and washed with distilled water, and approximately 2 g of harvested mycelium was frozen in liquid nitrogen and ground to a fine powder. DNA was extracted via the DNAeasy Plant Mini Kit (Qiagen, Hilden, Germany) following the manufacturer’s instructions with minor modifications. Briefly, mycelial material was harvested from actively growing cultures, disrupted by mechanical homogenization (FastPrep MP Biomedicals, Solon, OK, USA), and subjected to lysis using the AP1 buffer. After RNase A treatment and subsequent purification steps, DNA was bound to a silica membrane, washed with AW buffers, and eluted in nuclease-free water. DNA purity was measured via a DeNovix spectrophotometer (USA) and verified via gel electrophoresis on 0.8% agarose stained with SYBRSafe (Thermo Fischer Scientific, Waltham, MA, USA).

### 2.4. PCR Amplification

Amplifications were conducted in 25 μL reactions containing 1× PCR buffer, 2.5 mΜ MgCl_2_, 0.2 mM of each oligonucleotide, 200 μM dNTPs (Applied Biosystems, Foster City, CA, USA), 10–50 ng of DNA, and 1 U Taq polymerase (Roche, Basel, Switzerland).

The ITS region was amplified via the primers ITS1 (5′TCCGTAGGTGAACCTGCGG) and ITS4 (5′TCCTCCGCTTATTGATATGC), following the protocol of [14]. For the beta tubulin (*BT2*) partial region, amplification was carried out with the primers *BT2*-F (5′ GGYAACCARATHGGTGCYGCY) and BT2R (5′ACCCTCRGTGTAGTGACCCTTGGC). Calmodulin (*CAL*) partial region amplification was performed via the primers CL1 (5′GARTWCAAGGAGGCCTTCTC) and CL2A (5′TTTTTGCATCATGAGTTGGAC), following the procedure of O’Donnell et al., 2000 [15]. Finally, the elongation factor (*TEF*) partial region was amplified with the primers EF1-F (5′CTGAGGCTCGTTACCAGGAG) and EF1-R (5′CGACTTGATGACACCGACAG), according to Rodrigues et al., 2013 [16].

Amplifications were carried out in a T100 thermocycler (Bio-Rad Laboratories, Heracles, CA, USA), and the products were analyzed via gel electrophoresis on 1% agarose with SYBR Safe in 0.5× TBE buffer (45 mM Tris-Base, 45 mM boric acid, and 1 mM EDTA). Sequencing was performed by Psomagen Inc. (Rockville, MD, USA).

### 2.5. Phylogenetic Analysis

Phylogenetic analysis was performed with sequences from GenBank corresponding to the ITS, *BT2*, *CAL*, and *TEF* genes. Sequence data were edited via Geneious Prime (2025.2), and alignments were generated via MUSCLE [17] with default settings. Genetic relationships were investigated via phylogenetic analysis via neighbor-joining (NJ), the unweighted pair group method with arithmetic mean (UPGMA), and maximum likelihood (ML). Considering the Bayesian information criterion (BIC) and Akaike information criterion (AIC), the best model was estimated for each dataset. Phylogenetic trees were constructed in Geneious Prime, MEGA 12 [18], and Mr Bayes 3.2.7 [19]. The reliability of each node was assessed via the bootstrap resampling procedure (1000 replicates) [20]; gaps and missing data were not included in the analysis. Reference sequences of *S. schenckii* CBS 35936, *S. luriei* ATCC 18616, *S. globosa* FMR 8595, *S. mexicana* CBS 120341, and *S. chilensis* CBS 139891 and CBS 139890 were retrieved from GenBank and used to construct the alignment and phylogenetic trees [5,7,16,21]. The nucleotide (π) and haplotype (Hd) diversities were estimated via DnaSP software version 6 [22].

### 2.6. Bioinformatic Analysis

Genetic datasets derived from four loci (*CAL*, *BT2*, ITS, and *TEF*) were analyzed to investigate the molecular diversity of *Sporothrix* isolates from Latin America. The multilocus sequence alignments were concatenated into a single matrix for downstream analyses. Minimum spanning trees (MSTs) were constructed via the Prim algorithm [23] to visualize the shortest genetic paths among isolates, reflecting evolutionary distances and potential geographic structuring. Principal component analysis (PCA) was performed to reduce dataset dimensionality and reveal clustering patterns among samples on the basis of genetic variation across the concatenated loci [24,25]. In parallel, multidimensional scaling (MDS) was applied to assess the overall similarity among isolates via pairwise distance matrices [26].

To further explore genetic relatedness without imposing strict phylogenetic assumptions, self-organizing maps (SOMs) were generated via an unsupervised neural network approach [27]. SOMs were configured with map dimensions scaled heuristically to the square root of the sample size to optimize the resolution [28]. Visual inspection of clustering patterns in SOM aided in identifying genetically coherent subgroups within and across sampling regions.

All computational analyses were conducted via BioNumerics v7.6 (Applied Maths, Sint-Martens-Latem, Belgium), which enables the integration of diverse typing data and the application of consistent parameters across MST, PCA, MDS, and SOM visualizations.

## 3. Results

**Figure 1:**
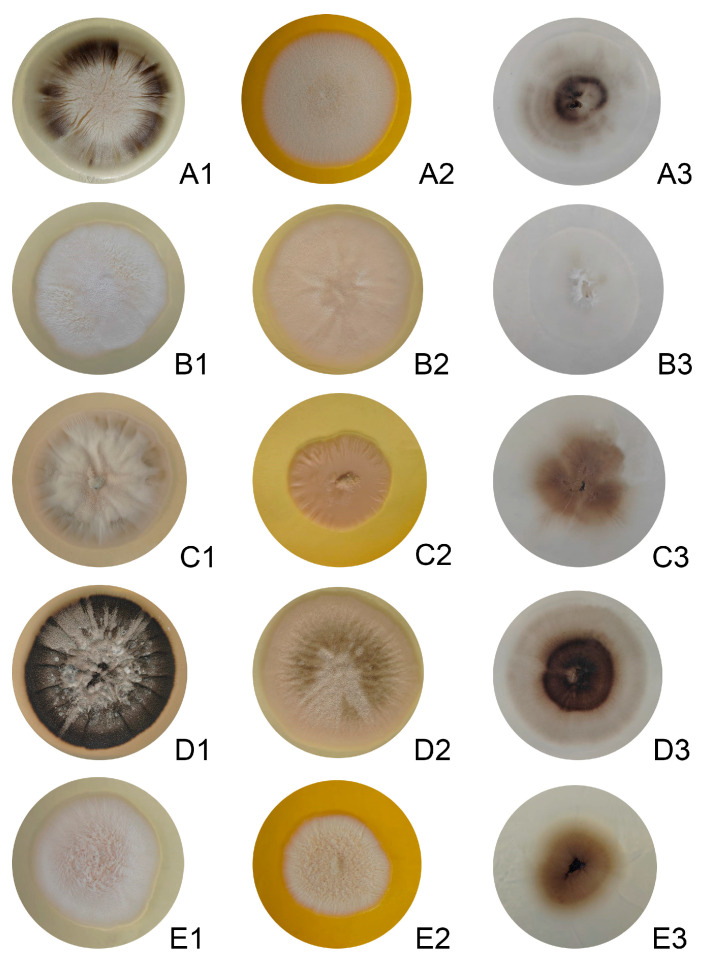
Colony morphology of *Sporothrix* isolates on different culture media. (**A1**–**A3**) EH194, (**B1**–**B3**) EH213, (**C1**–**C3**) EH257, (**D1**–**D3**) EH748, and (**E1**–**E3**) EH749. For each isolate, colonies are shown (from left to right) on potato dextrose agar (PDA), Sabouraud dextrose agar (SDA), and corn meal agar (CMA). Cultures were incubated at 28 °C for 7 days.

**Figure 2:**
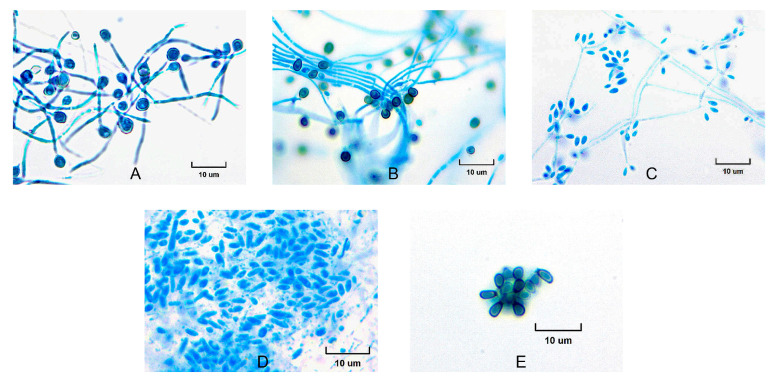
Microscopic morphology of *Sporothrix* conidia from isolates. (**A**) EH194, (**B**) EH213, (**C**) EH257, (**D**) EH748, and (**E**) EH749. Conidia were obtained from cultures grown on potato dextrose agar (PDA) for 7 days at 28 °C and stained with lactophenol cotton blue. Images were captured under light microscopy at 100× magnification.

The isolates exhibited a characteristic morphology, initially appearing white, with some developing brownish or nearly black colonies later (Figure 1). Microscopic examination revealed branched septate hyaline hyphae, and initially, simple egg-shaped conidia formed at the apex, which were arranged in a manner resembling a flower head (Figure 2). At 37 °C, the yeast cells were spherical, resembling blastospores; several buds appeared in the yeast cells. The yeast colony presented a creamy-colored surface. The dimensions of the conidia of the isolates ranged from 2 to 3 × 3 to 6 μm, whereas the yeast cell dimensions varied from 1 to 3 × 3 to 10 μm (refer to Table 1). All the isolates were positive in the transition to yeast test. With respect to the saccharose and raffinose assimilation tests, all the isolates presented positive results (Table 1).

The transition from white colonies to brownish or nearly black colonies, along with the presence of septate hyaline hyphae and egg-shaped conidia, is consistent with known pathogenic *Sporothrix* behavior under different temperatures and physiological conditions. At 37 °C, the isolates displayed a clear yeast phase with spherical blastospore-like cells, mirroring the budding observed at 25 °C. The uniformity in size range for conidia and yeast cells, along with the positive results in both the yeast phase transition and carbohydrate assimilation tests, aligns these morphological data with those of the clinical clade. Importantly, no distinctive or unique morphological features were identified in these isolates that would differentiate them from typical members of this clade.

The ITS datasets consisted of 598–752, *BT2* 469–659, *CAL* 1031–1045, and *TEF* 726–775 aligned positions. For the ITS analysis, the relationships were almost wholly unresolved. The major clades were poorly resolved via UPGMA, NJ, and ML (Supplementary Data S1).

The phylogenetic analysis using *CAL* sequences exhibited high phylogenetic resolution, particularly for distinguishing closely related species. The high bootstrap values indicate great reliability in resolving species-level relationships. The grouping of EH748, EH194 and EH257, as well as the grouping of EH213 and EH749 within a distinct phylogenetic subclade, suggests potential cryptic speciation (Supplementary Data S2).

The *TEF* tree shows a similar clustering pattern, although with moderate support values, suggesting that *TEF* might be less informative for resolving fine-scale species boundaries (Supplementary Data S3). Clustering persists across both loci, reinforcing the hypothesis that these strains may be undergoing incipient speciation. Branch lengths in the *CAL* tree suggest greater genetic divergence between the EH748-EH194-EH257 cluster and other *S. schenckii* strains, which could indicate reproductive isolation. Shorter branch lengths in *TEFs* imply conservation in housekeeping genes, which is expected in closely related cryptic species. The topologies of the *BT2* sequences were similar to those of the major clades (Supplementary Data S4).

**Figure 3:**
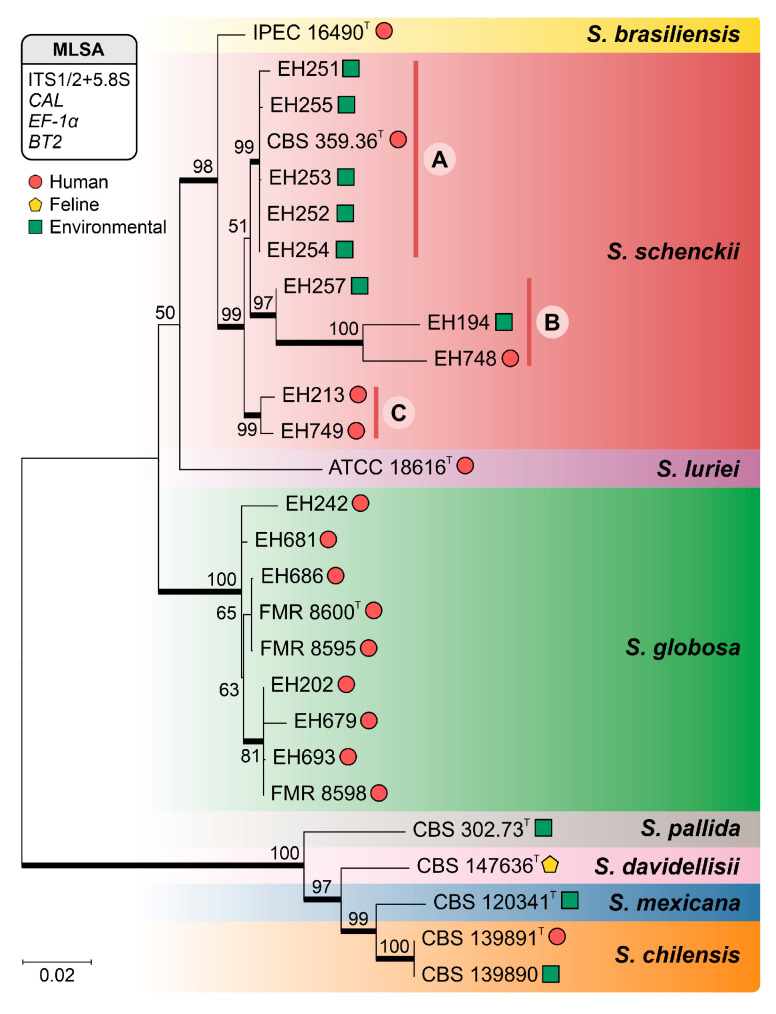
Phylogenetic analysis of *Sporothrix* isolates inferred by maximum likelihood. The tree was constructed on the basis of a concatenated alignment of the ITS, *CAL*, *BT2*, and TEF1-α gene fragments via the GTR+F+I+G4 model of nucleotide substitution. Bootstrap support values (>70%) from 1000 replicates are indicated at the nodes. The *S. schenckii* isolates were resolved into three distinct clades: clade A (CBS 359.36, EH254, EH252, EH253, EH251, and EH255), clade B (EH748, EH194, and EH257), and clade C (EH213 and EH749). The isolates are color-coded according to their respective MLSA clades. ^T^, a reference strain of each species.

Phylogenetic analysis of 2149 nucleotide sites resolved the *S. schenckii s. str.* isolates into three distinct clades. Clade A included CBS 359.36, EH254, EH252, EH253, EH251, and EH255. Clade B was composed of isolates EH748, EH194, and EH257, whereas clade C consisted of isolates EH213 and EH749 (Figure 3). The isolates EH202, EH242, EH679, EH681, EH686, and EH693 were initially identified as *S. schenckii* on the basis of phenotypic data. However, the subsequent phylogenetic analysis reassigned them to *S. globosa* (Figure 3). Notably, none of the sequences from the examined isolates were identified as belonging to *S. brasiliensis*, *S. luriei*, *S. chilensis*, *S. davidellisii* or *S. mexicana* (Figure 3).

With respect to the origin of the isolates, the trees indicate no strict ecological segregation. The placement of environmental isolates from Puebla alongside clinical strains from central Mexico and Guatemala reinforces the zoonotic or saprobic potential of the fungus, supporting the hypothesis of an active environmental reservoir.

A comparative analysis of genetic diversity among these clades revealed notable differences. *S. schenckii* clade C presented the greatest number of conserved characters (C = 1958), whereas clade B presented the greatest variability (V = 100). No parsimony-informative characters (Pi) were identified in any of the clades. The highest values for both singleton (S = 91) and nucleotide diversity (π = 0.03556) were observed in Clade B. In terms of haplotype analysis, Clade A presented the greatest number of haplotypes (H = 4). In contrast, clades B and C presented the highest haplotype diversity (Hd = 1.0). Furthermore, Clade B presented the maximum total number of mutations (η = 109) (Table 2). These findings indicate the presence of genetically distinct groups and underscore a discrepancy with the initial morphological identifications, which inaccurately classified all the isolates as *S. schenckii* (*s.l.*).

**Figure 4:**
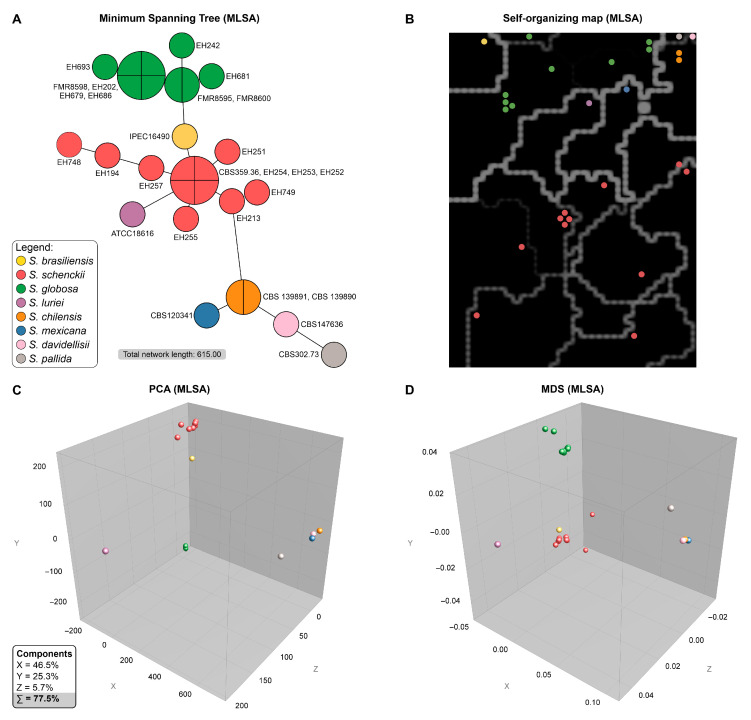
Genetic clustering of *Sporothrix* isolates on the basis of multilocus sequence analysis (MLSA). Genetic relationships among isolates were inferred from concatenated *CAL*, *BT2*, ITS, and *TEF* sequences. All methods consistently delineate species-level clusters. (**A**) Minimum spanning tree (MST) revealing that each node represents a unique genotype; edge lengths reflect genetic distances. The total network length (615.00) indicates overall diversity. (**B**) The self-organizing map (SOM) illustrates a clear separation of isolates into species-specific clusters. White boundary lines demarcate these major groups, highlighting the significant genetic distance between them. In contrast, thinner, darker lines reveal finer-scale variation within clusters, such as the intraspecific groups observed among the *S. schenckii* isolates (red). (**C**) Principal component analysis (PCA), which revealed a 3D distribution along the first three components, explaining 77.5% of the total variance. (**D**) Multidimensional scaling (MDS), providing an alternative spatial projection of genetic distances.

To further investigate the genetic structure, we employed four independent clustering methods: minimum spanning tree (MST), self-organizing maps (SOMs), principal component analysis (PCA), and multidimensional scaling (MDS). All analyses were performed on a concatenated multilocus alignment and yielded congruent, well-defined clustering patterns that were consistent with species-level identifications (Figure 4).

The MST analysis provided a clear visualization of the genetic distances among isolates, delineating *S. brasiliensis*, *S. schenckii*, *S. globosa*, and *S. luriei* as distinct groups that corresponded closely with recognized species boundaries (Figure 4A). A prominent cluster corresponding to *S. globosa* (green) included six isolates: EH202, EH242, EH679, EH681, EH686, and EH693. The majority of the EH isolates, however, were located within *S. schenckii* (red). Within this group, the MLSA clades were also recognized. Isolates from clade C (EH213 and EH749) were closely related, whereas isolates from clade A (EH254, EH252, and EH253) shared the same genotype as the type strain CBS 359.36 and were in close proximity to EH255 and EH251. The isolates from clade B (EH748, EH194, and EH257) were positioned close to each other and were distant from the members of clade A, suggesting significant genetic divergence and potential cryptic differentiation (Figure 3).

The SOM, which uses an unsupervised neural network algorithm, segregates the isolates into distinct spatial zones that mirror the species-level clusters observed in the MST (Figure 4B). The *S. globosa* isolates formed a compact, isolated territory with minimal diversity, whereas the *S. schenckii* subclades occupied separate subregions, reinforcing their genetic distinctiveness.

The PCA and MDS analyses, which reduce the dimensionality of the data for visualization, further corroborated these findings (Figure 4C,D). Both approaches revealed discrete, nonoverlapping clusters corresponding to *S. globosa* and *S. schenckii*. Within the *S. schenckii* group, the EH748/EH257/EH194 subgroup (clade B) consistently formed a peripheral satellite cluster, distinct from the central cluster comprising the other *S. schenckii* isolates (clades A and C). In the PCA, the first three principal components accounted for 77.5% of the total genetic variance, highlighting the robustness of the inferred genetic structure.

Collectively, the concordant results from the MST (Figure 4A), SOM (Figure 4B), PCA (Figure 4C), and MDS (Figure 4D) analyses demonstrated clear genetic differentiation between the *S. globosa* and *S. schenckii* isolates and revealed additional substructures within *S. schenckii*. The consistent placement of the EH isolates into these distinct clusters reinforces the hypothesis of ongoing diversification and potential cryptic speciation within the clinical clade of *Sporothrix*. Nevertheless, there was no apparent correlation between the genetic clusters and other data points, such as clinical form, patient sex/age, colony size, or cell dimensions, as these features varied across all groups.

## 4. Discussion

Sporotrichosis represents a significant and growing global health burden [29,30]. Initially recognized as a localized infection affecting individuals exposed to contaminated soil or plant material, sporotrichosis has expanded in both geographic range and severity and is now emerging as a zoonotic and endemic disease in various regions, especially Latin America [31] and parts of Asia [32]. This epidemiological shift, largely driven by the emergence of the highly virulent, cat-transmitted *Sporothrix brasiliensis*, underscores the critical need for molecular surveillance to understand the population structure, phylogeography, and evolutionary dynamics of pathogenic *Sporothrix* species [30]. Our study contributes to this effort by providing an MLSA of clinical and environmental isolates from Mexico, Guatemala, and Colombia, regions outside the primary zoonotic epicenter. We successfully resolved *S. schenckii s. str.* isolates into three distinct clades (A–C), identified isolates of *S. globosa*, observed an overlap between environmental and clinical genotypes, and, most notably, revealed a genetically divergent lineage (clade B) that suggests ongoing cryptic speciation within *S. schenckii s. str.* These findings provide a crucial snapshot of the *Sporothrix* population structure in Central America and northern South America, highlighting a different epidemiological landscape from that of the Southern Cone and reinforcing the complexity of this fungal pathogen group.

The primary contribution of this work is the robust molecular evidence for cryptic speciation within *S. schenckii s. str.* Our phylogenetic and population genetic analyses identified a well-supported monophyletic lineage, clade B, which includes both clinical (EH748) and environmental (EH194, EH257) isolates. This clade is characterized by significant genetic divergence from other *S. schenckii s. str.* isolates, as evidenced by its distinct clustering in PCA and MDS analyses and its high nucleotide diversity (π = 0.03556) and large number of singleton mutations. This discovery aligns with the broader paradigm in medical mycology, where many pathogenic fungi, once considered single species, have been resolved into complexes of cryptic species with distinct molecular, ecological, and sometimes clinical profiles [33]. The high resolving power of the *CAL* locus in our study is consistent with seminal MLST-based research that first established the *S. schenckii* species complex and demonstrated its utility as a core phylogenetic marker for the genus [1,13]. The patterns we observe are not merely intraspecific variation but are indicative of an active evolutionary process of diversification and lineage formation, a hallmark of incipient speciation [34].

This phenomenon of cryptic speciation is not unique to *Sporothrix* and is a recurring theme among thermally dimorphic fungal pathogens that navigate both environmental and mammalian host niches. For example, *Histoplasma capsulatum*, the agent of histoplasmosis, was historically considered a single species but has been definitively resolved by genomic data into at least four distinct species with strong phylogeographic signatures (*H. mississipiense*, *H. ohiense*, *H. suramericanum*, and *H. capsulatum s. str.*) [35,36]. Some of these lineages are associated with different clinical presentations, such as the distinct skin and bone involvement caused by African isolates, which are now recognized as part of a separate lineage [37,38]. Similarly, the agent of paracoccidioidomycosis, *Paracoccidioides brasiliensis*, has been shown to be a species complex comprising at least three cryptic lineages with a sympatric distribution, suggesting the presence of reproductive barriers other than geographic isolation [39]. The discovery of divergent clade B in our study is therefore consistent with this broader evolutionary pattern [6,29,40]. This finding suggests that, like *Histoplasma* and *Paracoccidioides*, *S. schenckii s. str.* is not a monolithic entity but rather a dynamic group of evolving lineages whose full diversity is only now being uncovered through molecular tools [4,5,30,41,42,43,44].

Our findings also provide critical phylogeographic data defining the epidemiological landscape of sporotrichosis in the studied regions. The identification of only *S. schenckii s. str.* and *S. globosa* and the conspicuous absence of *S. brasiliensis* are findings of major public health significance. The ongoing epizootic of cat-transmitted sporotrichosis in South America, driven by the hypervirulent *S. brasiliensis*, has been characterized by rapid geographic expansion from its epicenter in Rio de Janeiro, Brazil, to neighboring countries, including Argentina, Chile, and Paraguay, and has even been reported in imported cases in Europe and the United States [30,45,46,47]. The absence of *S. brasiliensis* in our cohort from Mexico, Guatemala, and Colombia strongly suggests that this southern epidemic has not yet reached these northern regions, implying the existence of a biogeographic or ecological barrier [9,11,30,48,49,50,51,52]. This contrasts with the epidemiology in Asia, where *S. globosa* is the predominant agent of classical, sapronotic sporotrichosis [32,53,54,55]. Our results thus confirm that the epidemiology of sporotrichosis in Central America and the northern Andes remains distinct from these other global hotspots, characterized by the circulation of *S. schenckii s. str.* and *S. globosa* [13,41,56,57].

Furthermore, the ecological overlap observed in our phylogenetic trees, where environmental isolates from soil cluster closely with clinical isolates, reinforces the classical sapronotic transmission model for *S. schenckii*. A *Sporothrix* sapronotic model posits that soil and plant matter are the primary reservoirs from which humans are infected via traumatic inoculation [58]. The tight genetic linkage between soil and human isolates, particularly within the divergent clade B, suggests that the traits driving the evolution of this lineage may be selected for in the environment. Virulence factors in *Sporothrix*, such as thermotolerance, melanin production, and protease secretion, are critical for survival in both soil and host tissue, suggesting that environmental adaptation may serve as a preadaptation mechanism for pathogenicity [59]. This ecological context is particularly relevant given the unresolved questions surrounding the origin of *S. brasiliensis*. While early genomic studies suggested a recent clonal expansion from a bottleneck population in Rio de Janeiro [30,46,60], more recent high-resolution genotyping has revealed immense genetic diversity within *S. brasiliensis* [30,46,61], estimating its origin more than 50,000 years ago and suggesting numerous independent zoonotic introductions from a yet-undiscovered environmental reservoir [62,63,64]. Understanding the environmental life cycle of *S. schenckii*, as our study did, may therefore provide crucial clues for identifying the cryptic reservoir of its more virulent sister species.

From a methodological standpoint, our study demonstrates the utility of a well-chosen MLSA scheme for species identification and the detection of intraspecific population structure [5,7]. The superior performance of protein-coding loci such as *CAL* and *TEF* over the universal fungal barcode ITS, which fails to resolve closely related isolates, is well documented and validates our approach [1,42]. However, these findings must be placed in the context of the current genomic era. While MLSA provides a robust framework, whole-genome sequencing (WGS) has emerged as the new gold standard for microbial epidemiology, offering unparalleled resolution [65,66,67,68]. Studies on other fungal pathogens have consistently shown that WGS can reveal fine-scale population structures, transmission pathways, and cryptic lineages that are invisible to MLSA. For example, a WGS study of Australian *Sporothrix* isolates provided significantly greater phylogenetic resolution than *CAL* sequencing alone [69]. The clear divergence of clade B, detected with just four loci, strongly implies that its genetic isolation would be even more pronounced at the whole-genome level. Therefore, our MLSA-based findings should be viewed as compelling, albeit likely conservative, estimates of the true genetic diversity present.

The identification of new clades suggests that *S. schenckii* is more genetically diverse than previously understood. The new clades may resolve ambiguities between species that are morphologically similar but genetically distinct, leading to more accurate classification. The *CAL* and *TEF* phylogenies both revealed strongly supported monophyletic clusters containing EH748, EH194, and EH257 within the clinical clade. Several lines of evidence suggest that this clade might represent cryptic species within *S. schenckii*. The discovery of this cryptic diversity has direct clinical implications. The recognized species in the clinical clade exhibit significant differences in virulence, with *S. brasiliensis* being more virulent in animal models than *S. schenckii*, which is in turn more virulent than *S. globosa* [70]. These species can also display different antifungal susceptibility profiles, with reports of reduced susceptibility and clinical resistance to itraconazole, the first-line therapy, becoming more frequent, particularly in the context of the *S. brasiliensis* epizootic [71,72]. The existence of a novel, genetically distinct lineage such as clade B raises the critical and testable hypothesis that it may also possess a unique clinical phenotype in terms of its intrinsic virulence or baseline antifungal susceptibility.

In acknowledging the limitations of our study, we note the relatively small sample size and the targeted, rather than exhaustive, geographic sampling. Our reliance on MLSA, while effective, provides a lower-resolution view than WGS does. Future studies should aim to characterize this clade at the genomic, phenotypic, and pathogenic levels. The highest priority is to apply WGS to isolates from clade B to definitively assess their taxonomic status and explore their genomic architecture for clues related to adaptation and virulence. Expanded molecular surveillance across Central America and northern South America is needed to determine the prevalence and geographic boundaries of these clades. Finally, linking these novel genotypes to phenotypes is essential. Comparative genomic analyses could elucidate gene content variations linked to virulence, environmental adaptation, or resistance mechanisms. Additionally, in vitro and in vivo pathogenicity assays could help determine whether this clade exhibits distinct clinical manifestations or tissue tropism. Epidemiological surveys would also be valuable for assessing its geographic distribution, reservoir hosts, and potential emergence as a public health concern. By integrating molecular, ecological, and clinical data, we can better understand the implications of this cryptic diversity and improve diagnostics, treatment strategies, and disease surveillance for sporotrichosis.

## 5. Conclusions

Our study provides robust evidence that the clinical clade encompasses cryptic species, as demonstrated through a polyphasic approach that integrates morphological assessment, culture-based characterization, and multilocus phylogenetic analysis of different loci. This analysis of clinical and environmental isolates led to the identification of a previously unrecognized clade within the complex. Notably, the *CAL* region proved to be the most informative marker for species-level resolution, whereas the *TEF* region served as a valuable secondary marker for confirming phylogenetic relationships within conserved clades. These findings refine the current taxonomy of the *Sporothrix* genus and underscore the critical role of molecular tools in revealing hidden fungal diversity, with direct implications for epidemiological surveillance, accurate diagnosis, and the development of targeted treatment strategies.

## Figures and Tables

**Table 1:** Description of clinical and environmental *Sporothrix* isolates.

						Genbank Accession Code				
ID	Origin	Source	Clinical Form	Sex	Age	ITS	*BT2*	*CAL*	*TEF*	Donated by
EH-194	Puebla, México	Soil	NA	NA	NA	PX275390				Universidad Autónoma de Puebla
EH-202	México	Human	L	M	28	PX275391				Centro Dermatológico Dr. Ladislao de la Pascua
EH-213	México	Human	F	M	47	PX275392				Centro Dermatológico Dr. Ladislao de la Pascua
EH-242	Guatemala	Human	U	U	U	PX275393				Universidad de San Carlos de Guatemala
EH-251	Puebla, México	Soil	NA	NA	NA	PX275394				Universidad Autónoma de Puebla
EH-252	Puebla, México	Soil	NA	NA	NA	PX275395				Universidad Autónoma de Puebla
EH-253	Puebla, México	Soil	NA	NA	NA	PX275396				Universidad Autónoma de Puebla
EH-254	Puebla, México	Soil	NA	NA	NA	PX275397				Universidad Autónoma de Puebla
EH-255	Puebla, México	Soil	NA	NA	NA	PX275398				Universidad Autónoma de Puebla
EH-257	Puebla, México	Soil	NA	NA	NA	PX275399				Universidad Autónoma de Puebla
EH-679	Colombia	Human	F	M	52	PX275400				Universidad Autónoma de Colombia
EH-681	Colombia	Human	L	M	17	PX275401				Universidad Autónoma de Colombia
EH-686	Colombia	Human	F	F	51	PX275402				Universidad Autónoma de Colombia
EH-693	Colombia	Human	L	F	18	PX275403				Universidad Autónoma de Colombia
EH-748	Guadalajara, México	Human	F	F	70	PX275404				Inst Dermatológico de Jalisco Dr. José Barba Rubio
EH-749	Puebla, México	Human	D	M	20	PX275405				Hospital General Dr. Manuel Gea Gonzalez

F, fixed; L, lymphocutaneous; D, disseminated. Sex: F, female; M, male; U, unknown.

**Table 2:** Genetic diversity indices of *Sporothrix schenckii* isolates based on concatenated loci (ITS, *TEF*, *CAL*, and *BT2*).

Clade	Isolates (*n*)	No. of Sites	C	V	Pi	S	π	H	Hd	Eta
A	5	2149	1924	9	0	9	0.00186	4	0.900	9
B	3	2149	1859	100	0	91	0.03556	3	1.0	109
C	2	2149	1958	14	0	14	0.00710	2	1.0	14

C: conserved characters; V: variable characters; Pi: parsimony-informative characters; S: singletons; π: nucleotide diversity; H: haplotype number; Hd: haplotype diversity; Eta: total number of mutations.

## Data Availability

The original contributions presented in this study are included in the article/Supplementary Material. Further inquiries can be directed to the corresponding author.

## Supplementary Materials

The following supporting information can be downloaded at https://www.mdpi.com/article/10.3390/jof11110759/s1, Supplementary Data S1: Phylogenetic Trees Based on ITS Sequences Constructed by UPGMA, NJ, and ML Methods; Supplementary Data S2: Phylogenetic Trees Based on *CAL* Sequences Constructed by UPGMA, NJ, and ML Methods; Supplementary Data S3: Phylogenetic Trees Based on *TEF* Sequences Constructed by UPGMA, NJ, and ML Methods; Supplementary Data S4: Phylogenetic Trees Based on *BT2* Sequences Constructed by UPGMA, NJ, and ML Methods.
