# Supplementary figures and images for: Cryptic Diversity and Ecological Overlap in Sporothrix schenckii: Insights from Multilocus Phylogenetics of Clinical and Environmental Isolates
Source: J Fungi (Basel). 2025 Oct 22;11(11):759. doi: 10.3390/jof11110759 (PMC12653030; doi:10.3390/jof11110759)

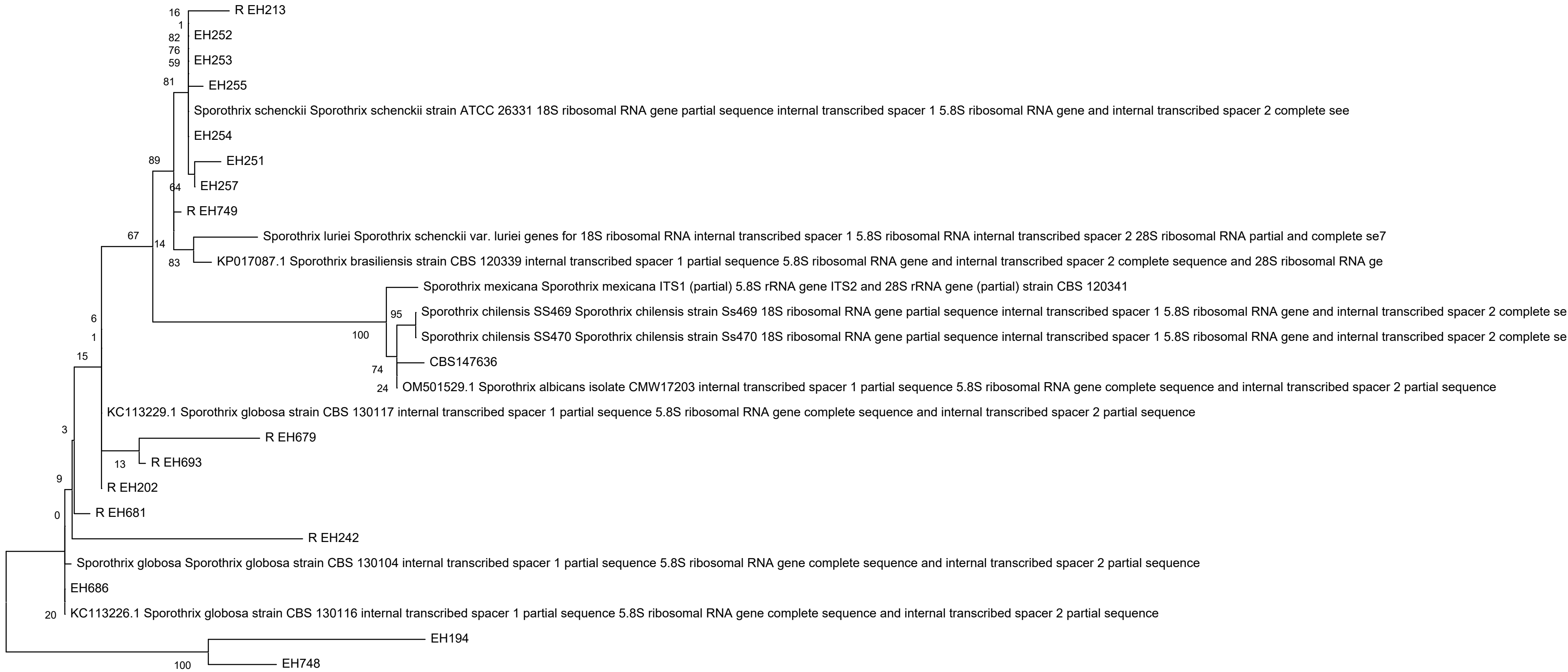

0.02

Supplement: Supplementary file 1 [file jof-11-00759-s001.zip › Supplementary_files/Supplementary_Data_1/SD_ITS_MV_SPORO.pdf]

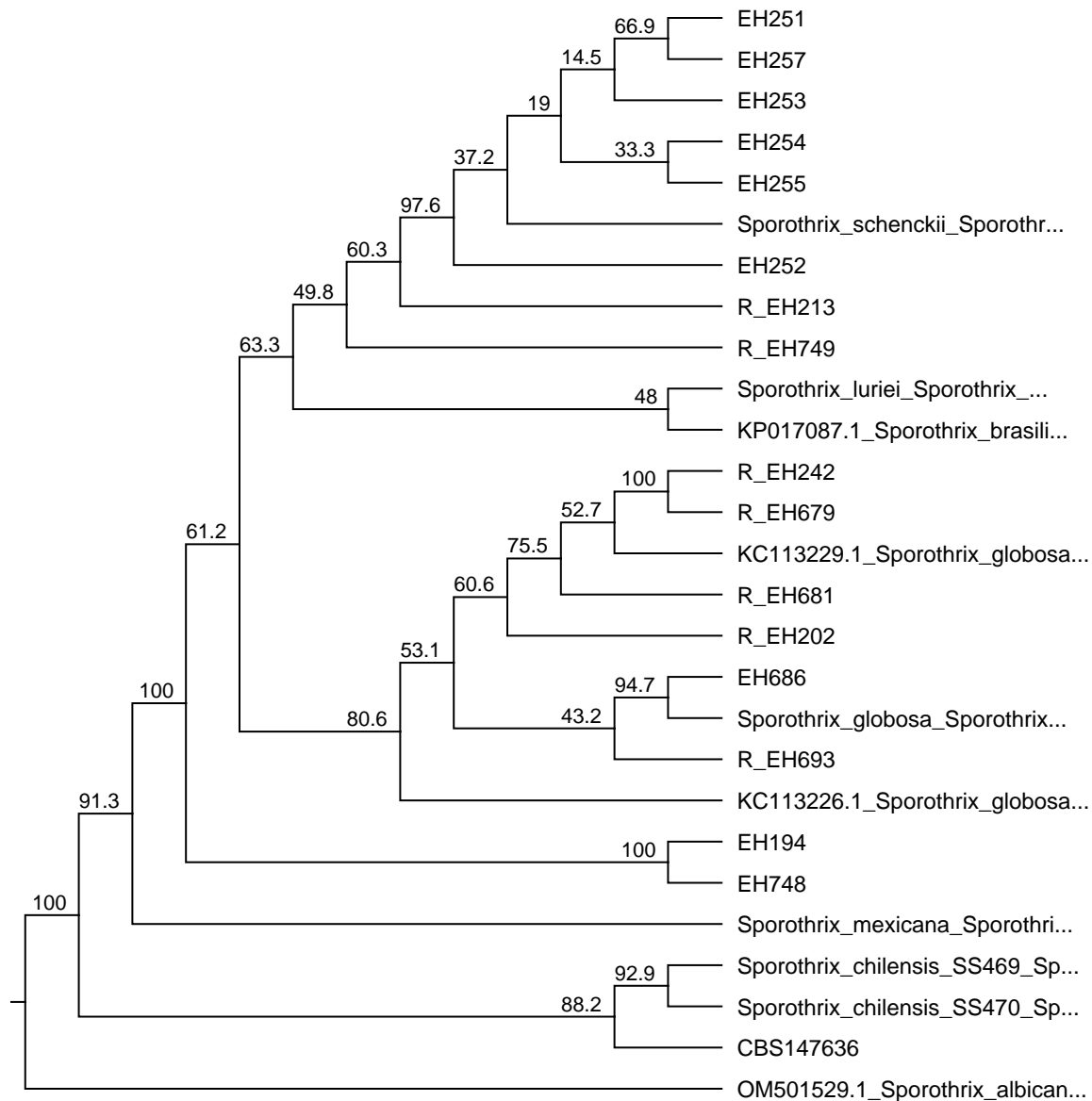

Supplement: Supplementary file 1 [file jof-11-00759-s001.zip › Supplementary_files/Supplementary_Data_1/SD_ITS_NJ_SPORO.pdf]

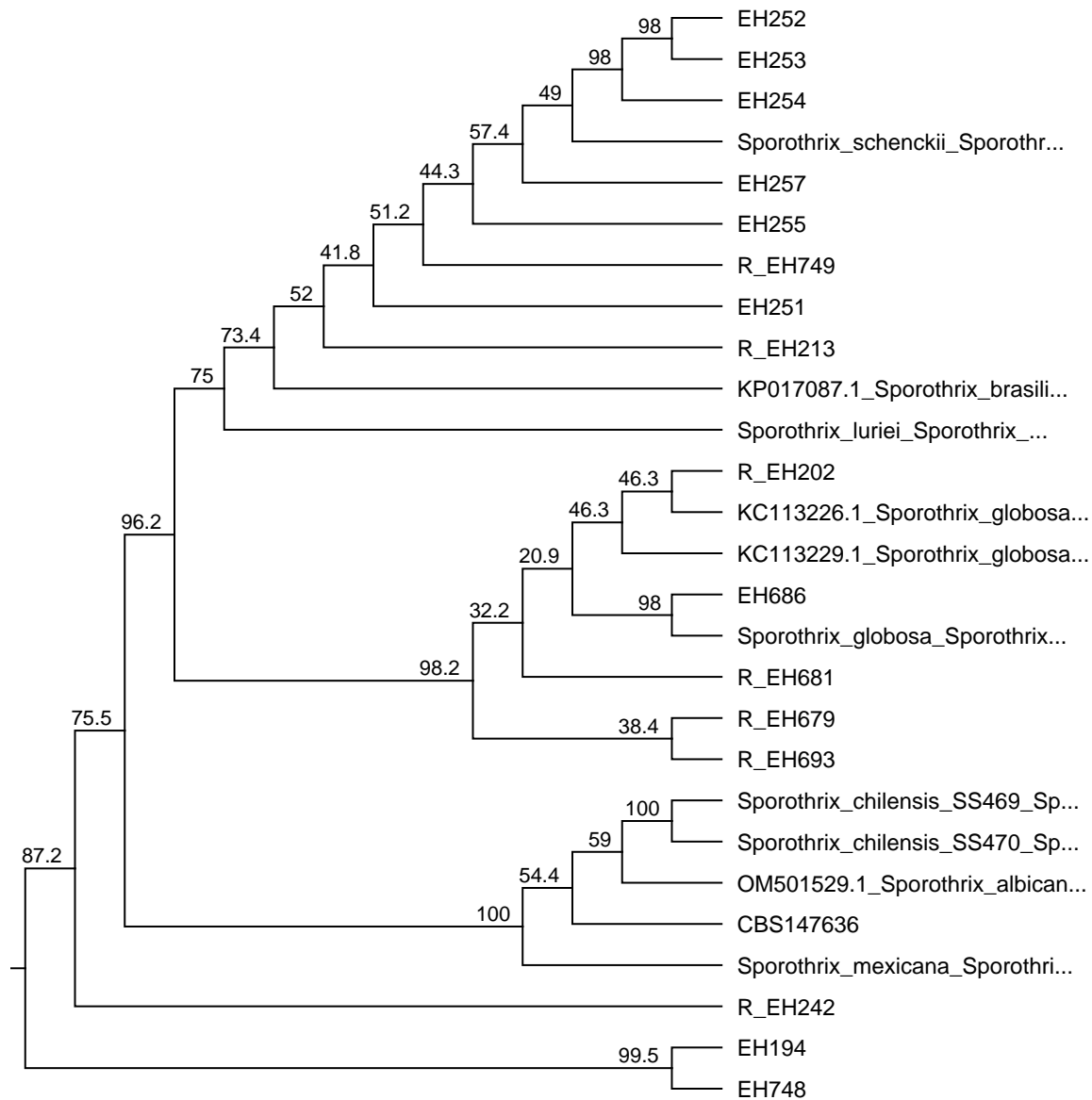

Supplement: Supplementary file 1 [file jof-11-00759-s001.zip › Supplementary_files/Supplementary_Data_1/SD_ITS_UPGMA_SPORO.pdf]

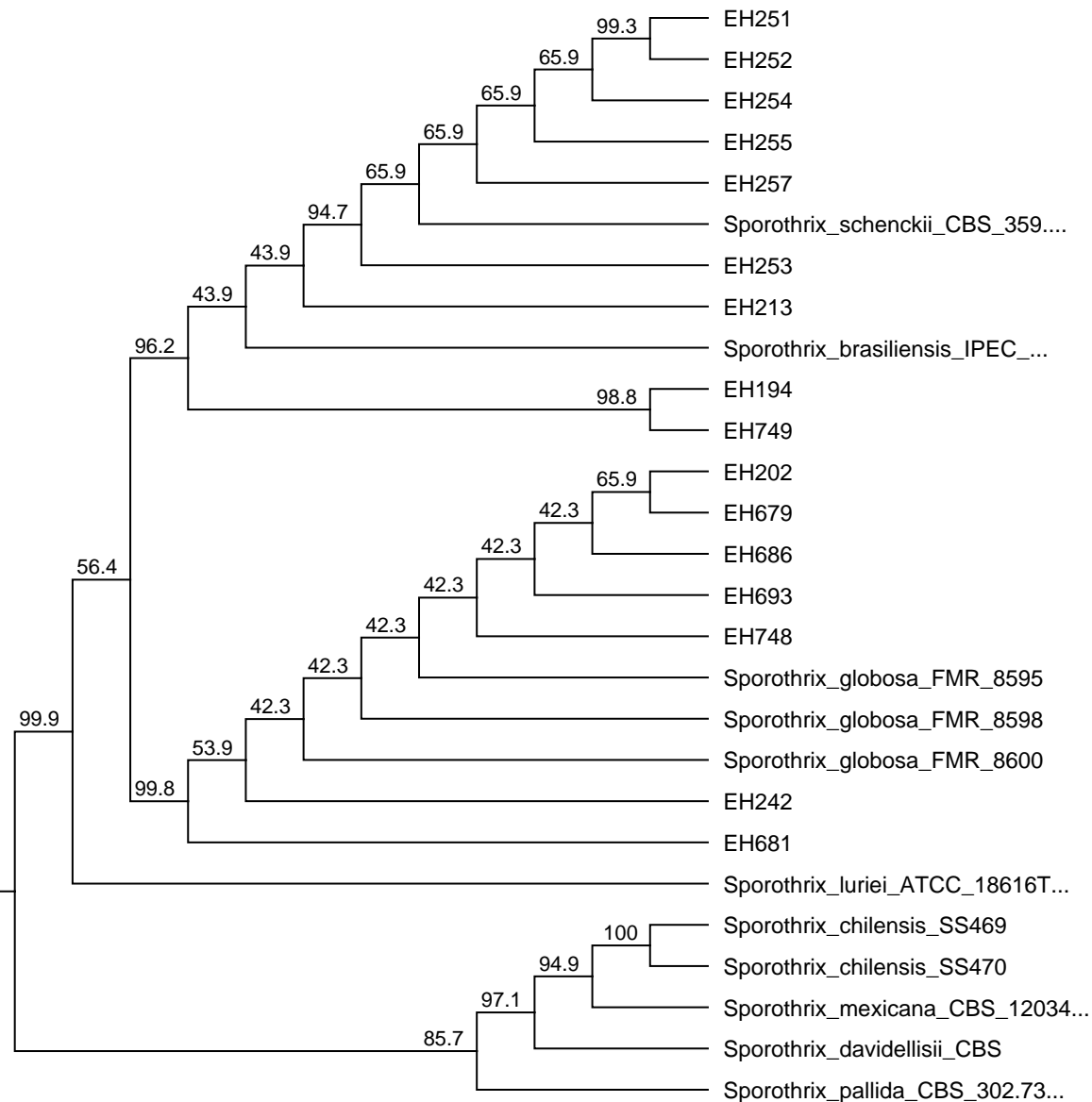

Supplement: Supplementary file 1 [file jof-11-00759-s001.zip › Supplementary_files/Supplementary_Data_4/SD_BTUB_UPGMA_SPORO.pdf]

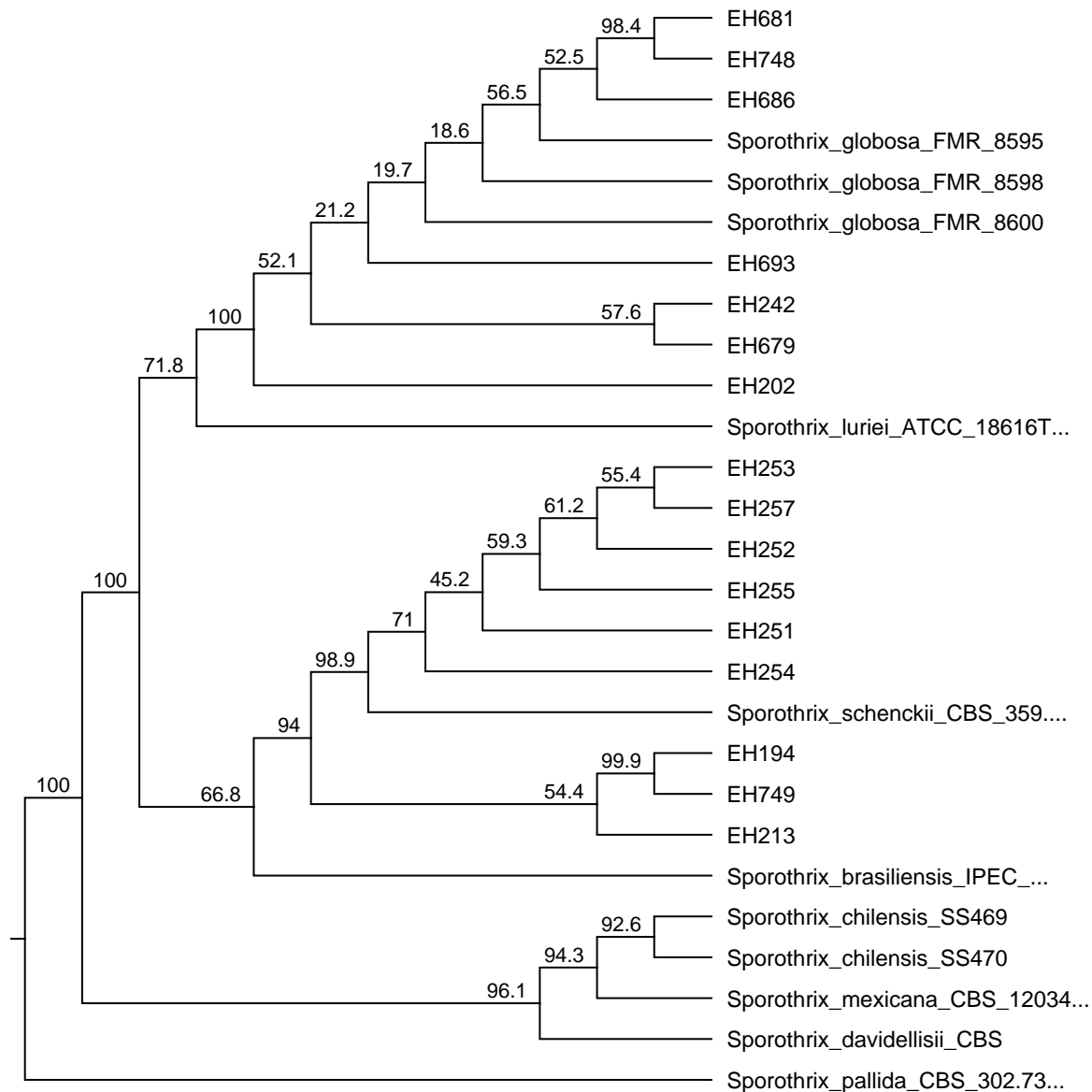

Supplement: Supplementary file 1 [file jof-11-00759-s001.zip › Supplementary_files/Supplementary_Data_4/SD_BTUB_NJ_SPORO.pdf]

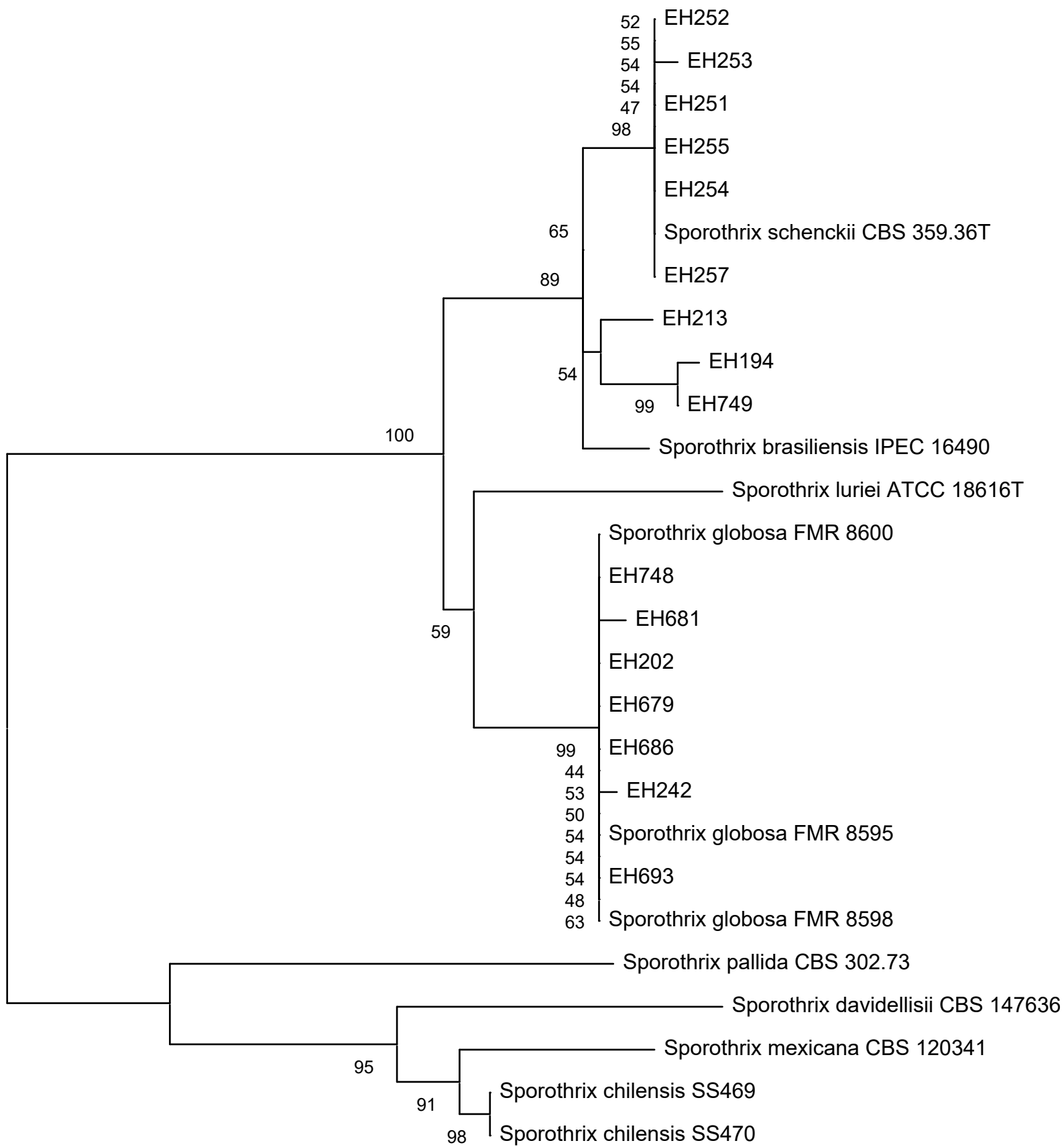

0.02

Supplement: Supplementary file 1 [file jof-11-00759-s001.zip › Supplementary_files/Supplementary_Data_4/SD_BTUB_MV_SPORO.pdf]

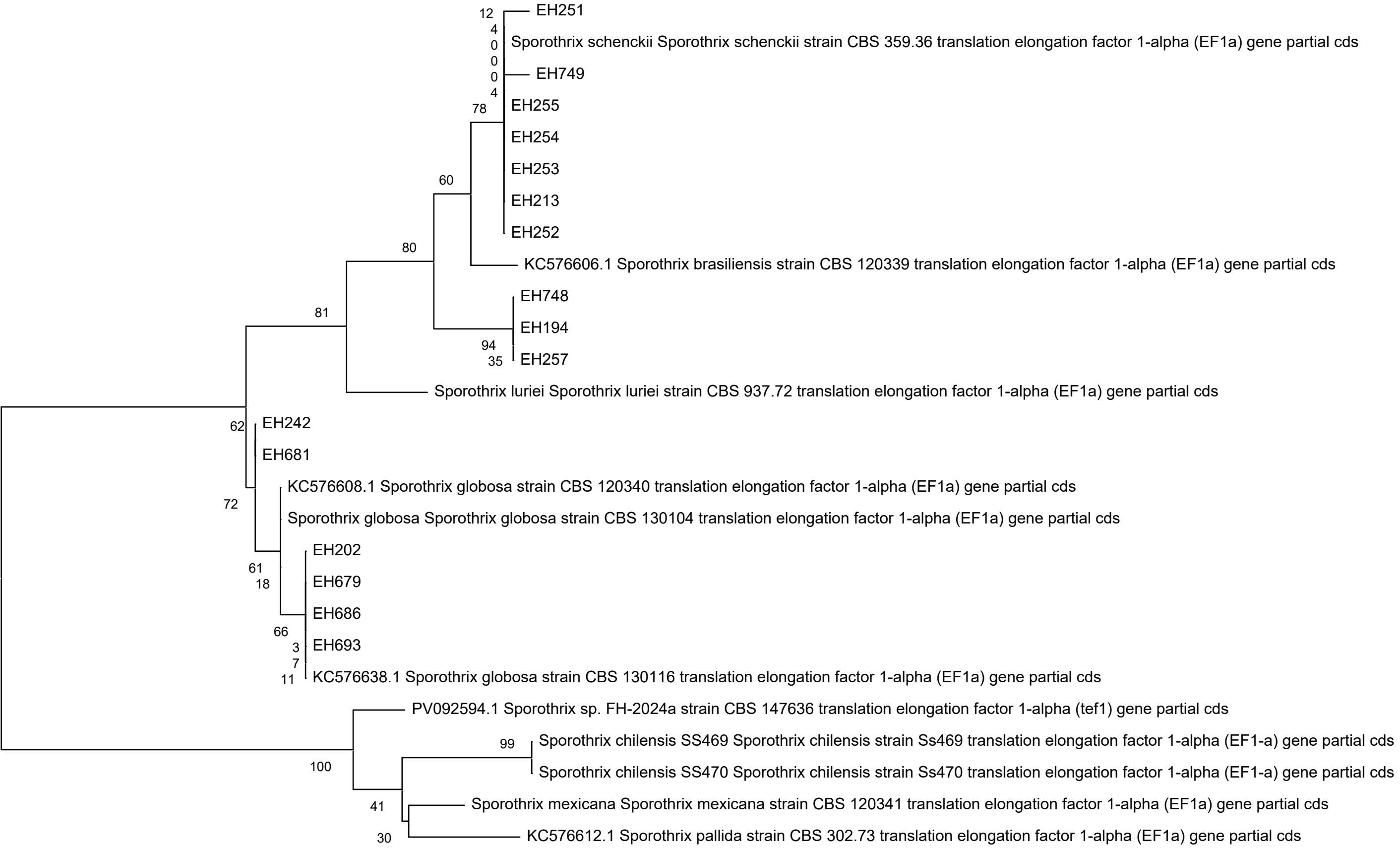

0.01

Supplement: Supplementary file 1 [file jof-11-00759-s001.zip › Supplementary_files/Supplementary_Data_3/SD_TEF_MV_SPORO.pdf]

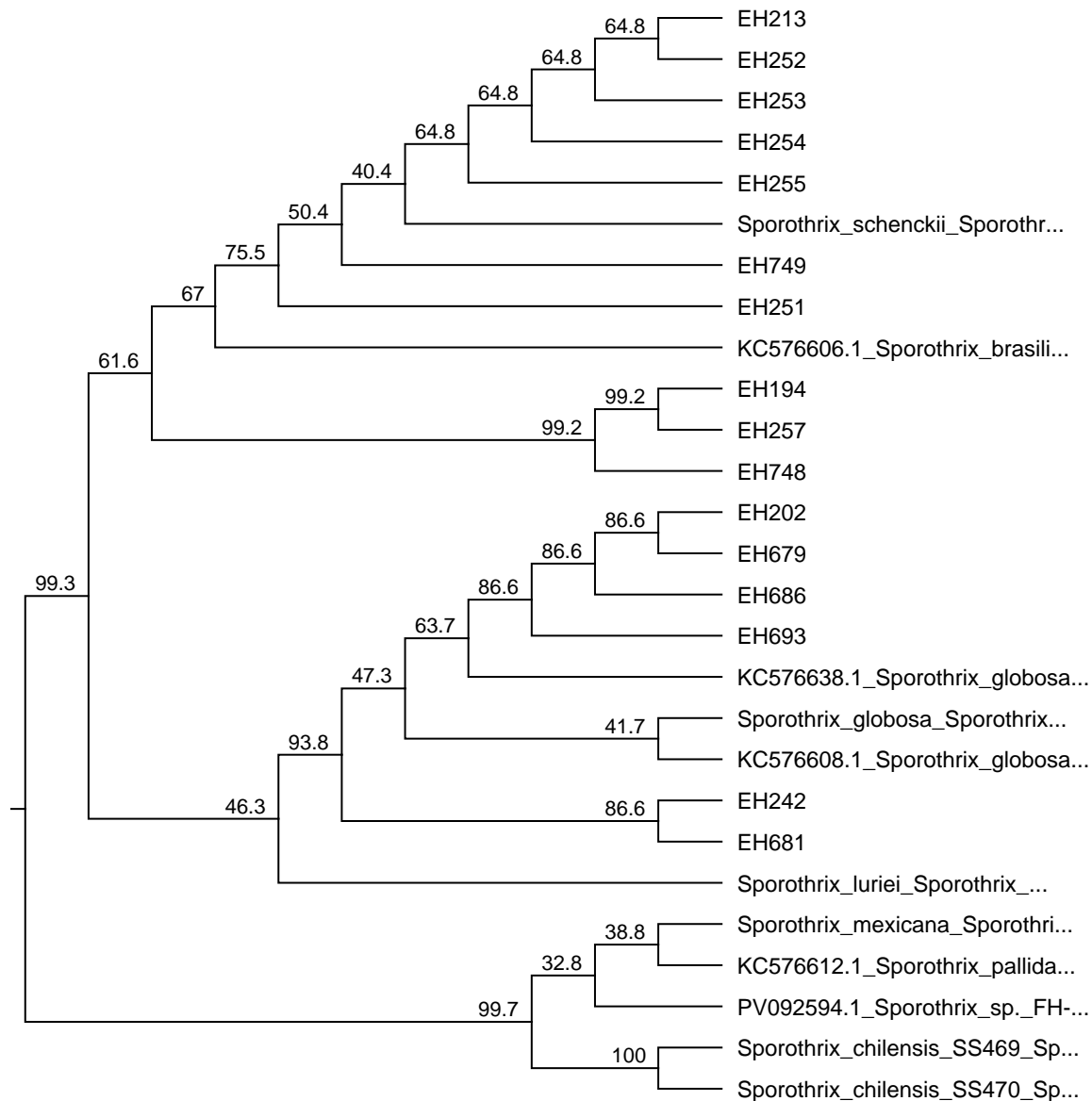

Supplement: Supplementary file 1 [file jof-11-00759-s001.zip › Supplementary_files/Supplementary_Data_3/SD_TEF_UPGMA_SPORO.pdf]

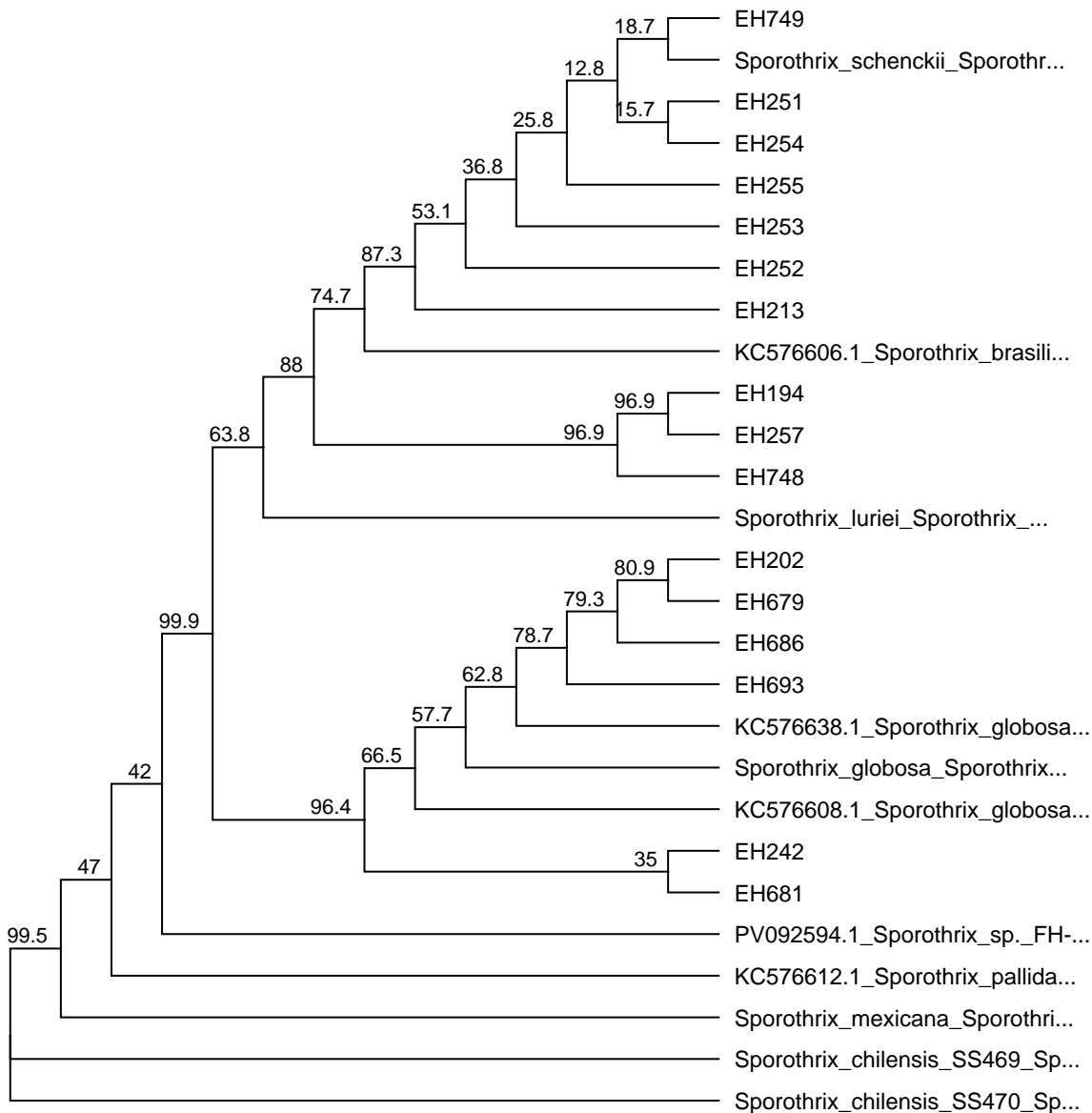

Supplement: Supplementary file 1 [file jof-11-00759-s001.zip › Supplementary_files/Supplementary_Data_3/SD_TEF_NJ_SPORO.pdf]

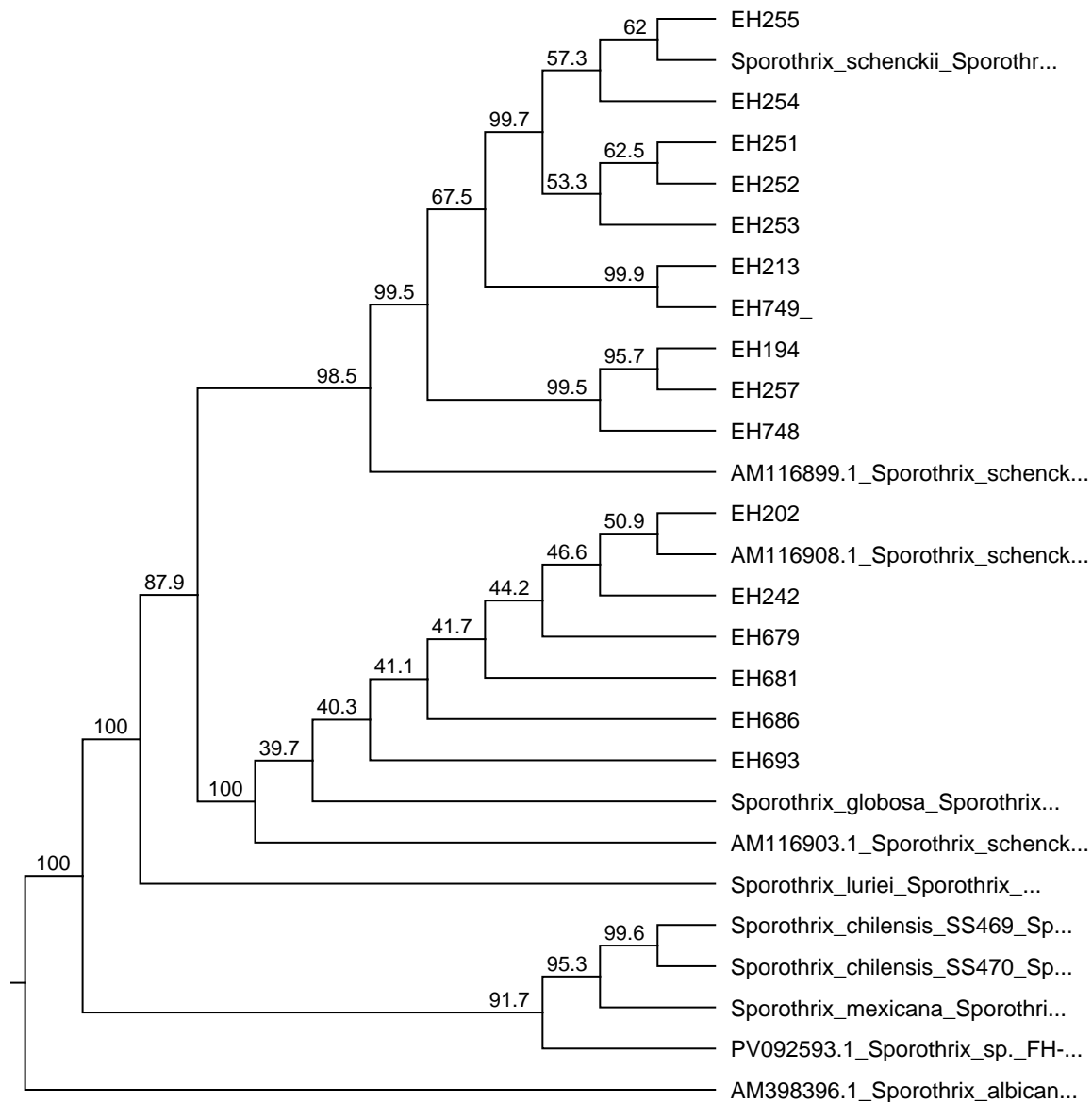

Supplement: Supplementary file 1 [file jof-11-00759-s001.zip › Supplementary_files/Supplementary_Data_2/SD_CAL_NJ_SPORO.pdf]

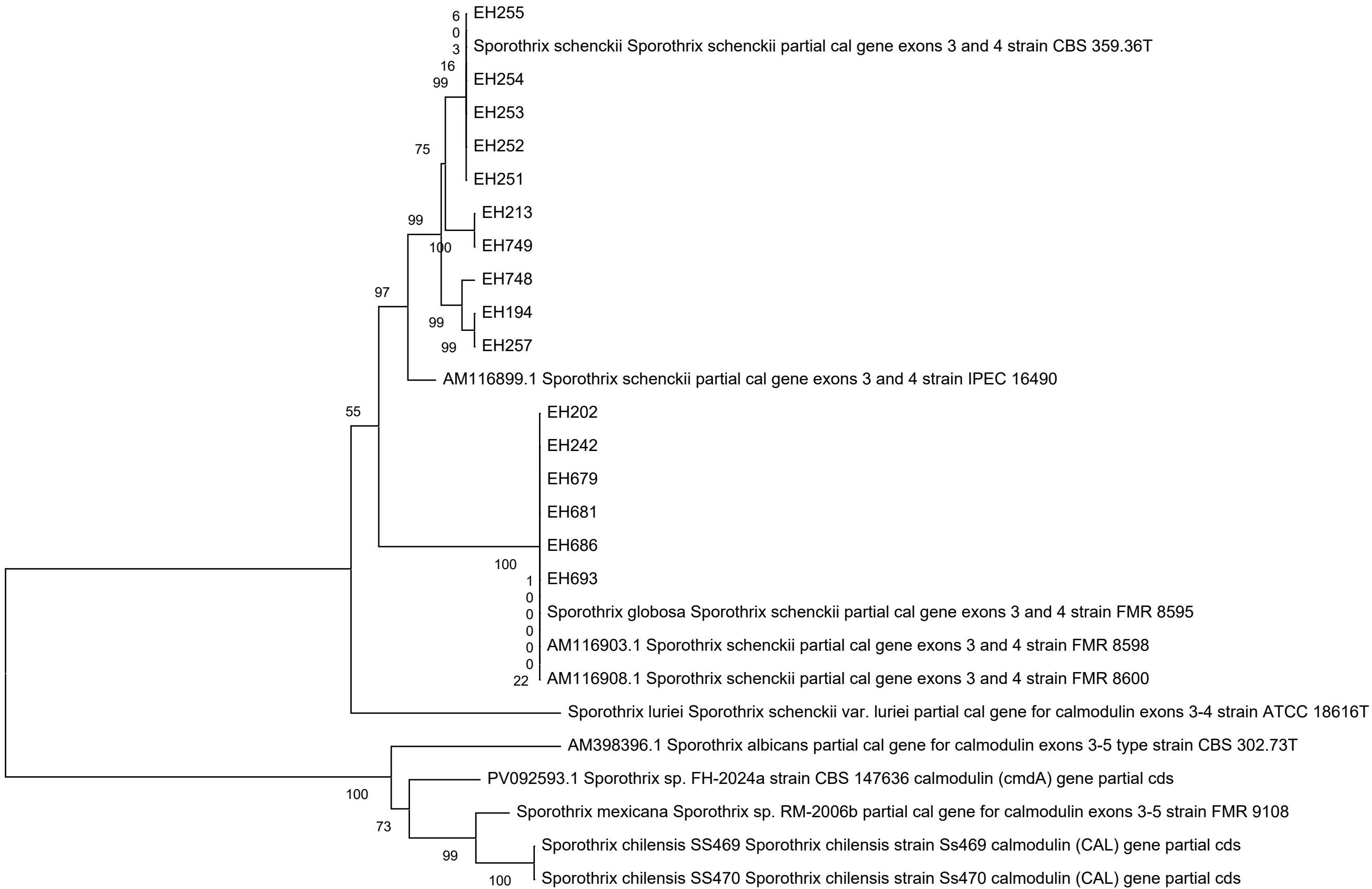

0.050

Supplement: Supplementary file 1 [file jof-11-00759-s001.zip › Supplementary_files/Supplementary_Data_2/SD_CAL_MV_SPORO.pdf]

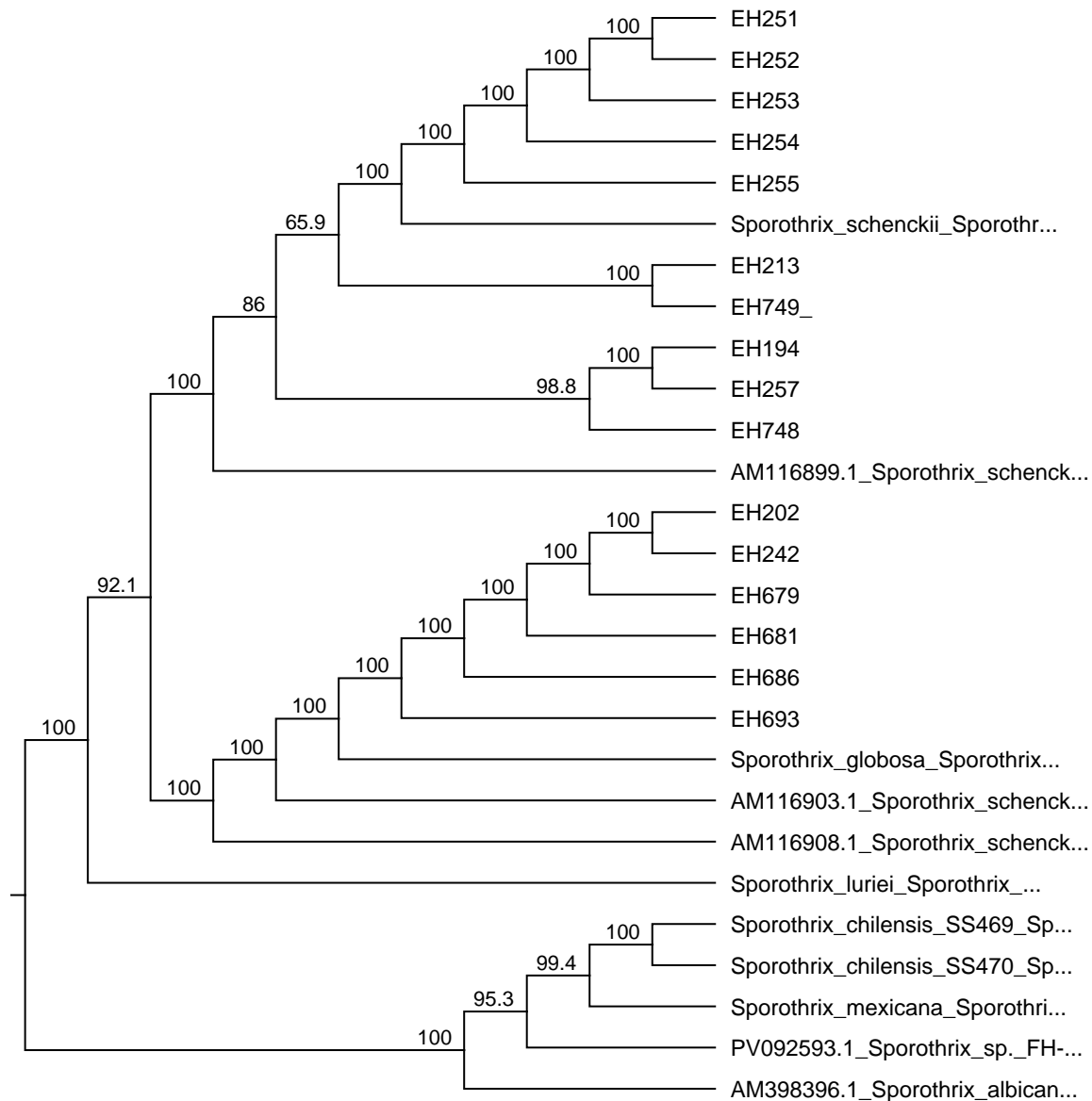

Supplement: Supplementary file 1 [file jof-11-00759-s001.zip › Supplementary_files/Supplementary_Data_2/SD_CAL_UPGMA_SPORO.pdf]
